# Analysis of the Acid Detergent Fibre Content in Turnip Greens and Turnip Tops (*Brassica rapa* L. Subsp. *rapa*) by Means of Near-Infrared Reflectance †

**DOI:** 10.3390/foods8090364

**Published:** 2019-08-26

**Authors:** Sara Obregón-Cano, Rafael Moreno-Rojas, Ana María Jurado-Millán, María Elena Cartea-González, Antonio De Haro-Bailón

**Affiliations:** 1 Department of Plant Breeding, Institute for Sustainable Agriculture (CSIC), 14004 Córdoba, Spain; 2 Department of Food Science and Technology, University of Córdoba, Rabanales Campus, 14014 Córdoba, Spain; 3 Department of Plant Genetics, Biological Mission of Galicia (CSIC), 36143 Pontevedra, Spain

**Keywords:** *Brassica rapa*, turnip greens, turnip tops, acid detergent fibre, NIRS

## Abstract

Standard wet chemistry analytical techniques currently used to determine plant fibre constituents are costly, time-consuming and destructive. In this paper the potential of near-infrared reflectance spectroscopy (NIRS) to analyse the contents of acid detergent fibre (ADF) in turnip greens and turnip tops has been assessed. Three calibration equations were developed: in the equation without mathematical treatment the coefficient of determination (*R*^2^) was 0.91, in the first-derivative treatment equation *R*^2^ = 0.95 and in the second-derivative treatment *R*^2^ = 0.96. The estimation accuracy was based on RPD (the ratio between the standard deviation and the standard error of validation) and RER (the ratio between the range of ADF of the validation as a whole and the standard error of prediction) of the external validation. RPD and RER values were of 2.75 and 9.00 for the treatment without derivative, 3.41 and 11.79 with first-derivative, and 3.10 and 11.03 with second-derivative. With the acid detergent residue spectrum the wavelengths were identified and associated with the ADF contained in the sample. The results showed a great potential of NIRS for predicting ADF content in turnip greens and turnip tops.

## 1. Introduction

The plants of the genus *Brassica* constitute one of the economically most important plant groups in the world. They are valuable sources of roots, stems, leaves, shoots and inflorescences, as well as of oils, condiments and forage for nutrition or industrial use [1]. Depending on the part of the plant used, these crops are classified as being oleaginous, forage, horticultural products and condiments. The growing scientific interest in this botanical group has increased in parallel to its economic importance and recent achievements in investigation. The consumption of vegetables of the genus *Brassica* has been related to human health with regard to the reduction in the risk of suffering from certain types of chronic diseases, such as cardiovascular problems and different types of cancer [2,3]. Within the genus *Brassica*, four species, *Brassica oleracea*, *Brassica rapa*, *Brassica napus* and *Brassica juncea*, are the crops with a horticultural use. *Brassica rapa* L. subsp. *rapa*, commonly known as turnip, is one of the oldest crops used for human consumption. It was the first species of *Brassica* domesticated by humans thousands of years ago, and it was already cited in Sanskrit literature under the name of Siddharta, which proves the antiqueness of its cultivation [4]. In the north of Spain and Portugal turnip greens and turnip tops are rising in value and they occupy a prominent place in traditional Galician and Portuguese agriculture. Turnip greens are the young leaves of turnips harvested in their vegetative period, whereas turnip tops are the floral stalks collected just before the flower opens. In the case of turnip tops, the diversification of this product is acquiring special importance, and the number of firms packing and freezing it is increasing, not only in Galicia but also in other parts of Spain. An important factor to be taken into account in the nutritional composition of both turnip tops and turnip greens is their fibre content, in addition to the presence of other components like some vitamins and minerals which partly complement the daily dietary demands. The fibre content in vegetables is essential to the digestibility of the food. It has been recognized that the ingestion of fibre is of great benefit to human health, contributing to the prevention of cancer of the colon and reducing the risk of developing cardiovascular diseases, cerebral infarction, hypertension, diabetes, obesity and certain gastrointestinal complaints [5]. Traditionally, the structural carbohydrates of foodstuffs have been estimated via the analysis of their crude fibre content. Crude fibre can be defined as being the residue resulting from submitting the food to a double hydrolysis: acid (with sulphuric acid) and alkaline (with potassium hydroxide), using the protocol developed by the Weende method [6]. One drawback of double hydrolysis is that it solubilizes part of the hemicellulose and of the lignin of the cell wall, so that the result obtained of the crude fibre content is lower than the real content in structural carbohydrates. This problem is avoided by using detergent solutions for the fibre analysis, following the method proposed by Goering and Van Soest [7]. Neutral detergent fibre (NDF) estimates the content in cellulose, hemicellulose, lignin, cutine and insoluble minerals in the cell wall, and is determined as being the residue remaining after extraction with the neutral detergent solution (made up of sodium lauryl sulphate and EDTA). Acid detergent fibre (ADF) is an estimator of the content in cellulose, lignin, cutine and insoluble minerals in the cell wall and it is determined as the residue remaining after the digestion of the sample with an acid detergent solution (made up of diluted sulphuric acid and cetyl-trimethyl-ammonium bromide). The difference between NDF and ADF is the fraction of hemicellulose. With the ADF method the hemicellulose is hydrolysed so that the determination of ADF is more closely associated with degradability and digestibility, whereas the NDF content is only related to ingestion or to a fraction of fibre still highly usable by the organism [8]. Several authors have documented the negative correlation existing between the content of NDF and ADF with the digestibility of vegetable products [9,10]. In the same sense, the high negative correlation between the ADF content and digestibility in vitro has been demonstrated, therefore, the ADF content in a vegetable could be considered as being a good indicator of its digestibility and quality [11,12,13].

Standard wet chemistry analytical techniques currently used to determine plant fibre constituents (as those described above) are costly, time-consuming and destructive. Additionally, they need specialized workers for their application. During the last 40 years technology based on near-infrared reflectance spectroscopy (NIRS) has become one of the most attractive analytical techniques that is routinely used to estimate numerous quality components in agriculture and food research, since analysis can be carried out at a low cost, with an important saving of time, and without using hazardous chemicals. Moreover, NIRS is a non-destructive technique which requires minimal or zero sample preparation [14,15,16,17,18,19,20]. Nowadays, NIRS technology is applied routinely in plant breeding programs for many vegetable species to determine their content in fibre, moisture, oil, protein, minerals, glucosinolates and fatty acid composition of their edible parts [21,22,23,24]. The first calibrations for the crude fibre content in seeds in the genus *Brassica* were carried out by Panford, Williams and Man [25] and Michalski, Ochodzki and Cicha [26]. More recently, calibrations for ADF in seeds of different *Brassica* species have been performed by Font, Del Río, Fernández and De Haro-Bailón [27], Font et al. [16], Dimov, Suprianto, Hermann and Möllers [28] and Wittkop, Snowdon and Friedt [29]. Lately, the NIRS technique has been used for the rapid determination of the quality of crude matter starting from the study of fibre as a component of biomass [30], in order to determine the digestibility of cane sugar [31], or to study the fibre content in food for ruminants [32]. 

This work has aimed to develop and validate NIRS calibration equations for the determination of acid detergent fibre (ADF) in aerial edible parts of *Brassica rapa* (turnip greens and turnip tops), in order to employ them as a tool for a fast and non-destructive analysis in the screening of germplasm and in the selection of genotypes of the highest quality with respect to this component.

## 2. Materials and Methods 

### 2.1. Plant Material 

During the seasons 2013–2014 and 2014–2015, a set of five varieties of *Brassica rapa* L. subsp. *rapa* were grown on the Institute for Sustainable Agriculture experimental farm in Córdoba, (37°51′ N, 4°48′ W, Spain) in a random block design with three replications. The climate is a typical Mediterranean one with a mean rainfall of 650 mm and deep loamy-sandy soil classified as Typic Xerofluent.

**Figure 1:**
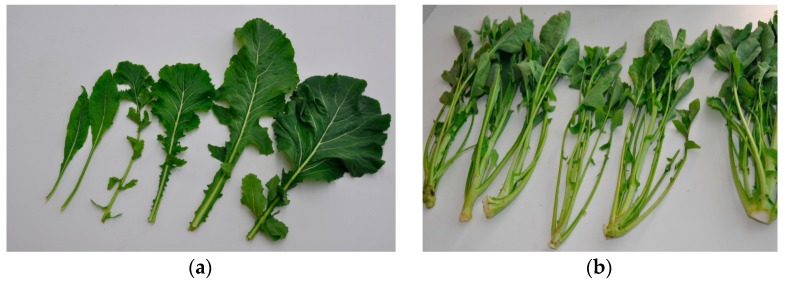
(**a**) Turnip greens; (**b**) turnip tops.

The *Brassica rapa* L. subsp. *rapa* varieties came from the *Brassica* Germplasm Bank at the Biological Mission of Galicia (Pontevedra), where they had been characterized by their agronomic characteristics and their aptitude for the production of turnip greens and turnip tops. During each agricultural season, and at the optimal consumption moment, samples of turnip greens (4–5 leaves per plant) and of turnip tops (3–4 flower stalks per plant) were harvested from the plants selected for each of the varieties studied (Figure 1). In total, 134 samples were harvested, 78 in the 2013–2014 season (34 turnip greens and 44 turnip tops) and 56 samples in the 2014–2015 season (29 turnip greens and 27 turnip tops). All the vegetable material was thoroughly washed with tap water to remove dirt and dust from its surface and, finally, it was rinsed with deionized water. Next, it was stored at −80 °C until its lyophilisation, which was done in Telstar^®^ model Cryodos-50 (Telstar, Terrasa, Spain) equipment. The lyophilized samples were ground in an IKA-Labortechnik^®^ (Staurfen, Germany) model A10 mill for 20 s and stored in desiccators up to the moment of being analysed by the reference method or scanned in the NIRS equipment.

### 2.2. Analysis of Acid Detergent Fibre

The ADF content was determined following the procedures described by Goering and Van Soest [7] in a Dosi-Fibre (Selecta^®^, Barcelona, Spain) machine. 0.5 g of lyophilized sample was weighed in glass filtering crucibles (porosity 2). This was digested for one hour in 100 mL of hot cetyl-methyl-ammonium bromide in an acid medium (sulphuric acid) and then filtered to obtain the residue considered as being the acid detergent fibre of the sample. Next, the residue was washed with hot water and acetone and dried in a stove at 110 °C for 90 min. Then it was stored in desiccators for 30 min to temper the crucibles and prevent the sample from becoming moist, after which the sample was weighed. The acid detergent residue (ADR) remaining after digestion was removed from the crucibles and stored to obtain the NIRS spectrum from the pure residue.

The acid detergent fibre of the sample was calculated according to Equation (1):

(1) $$\mathrm{ADF}\;\left(\%\right)=\frac{P3-P1}{P2-P1}\times 100,$$

where P1 is the crucible weight, P2 is the weight of the crucible with the sample and P3 is the weight of the crucible with the acid detergent residue after digestion. Each sample was analysed in duplicate.

### 2.3. Development of NIRS Equations

Sample spectra were recorded with a Model 6500 (Foss-NIRSystems^®^, Inc., Silver Spring, MD, USA) near-infrared spectrophotometer in the reflectance mode. One spectrum was recorded for each sample. The samples were placed in a round capsule 3 cm in diameter made of quartz glass and anodized aluminium to prevent interferences in their absorption. From each sample, reflectance spectra in the wavelength range of 400–2500 nm, at 2 nm intervals, were obtained. Collection of spectral data and their chemometric analysis was conducted with the WinISI II v1,50 software (Infrasoft International, Port Matilda, PA, USA). 

The spectral outliers were detected by a principal component analysis (PCA) applied to the whole set of the population based on the calculation of the Mahalanobis distance (H) [33,34]. In addition to being a tool for the selection of samples from the calibration set, this is a highly useful technique in the analysis for converting original spectra data (absorbance values) into new orthogonal variables (principal components) thus eliminating collinearity (redundant information) [35]. The CENTER algorithm included in the WinISI II software (version 1.50, Infrasoft International, Port Matilda, PA, USA) was used to calculate the H distances between the spectra of the different samples with respect to the mean spectrum. In agreement with the work of Shenk and Westerhaus [33], samples with a statistical H value of over three units were defined as being atypical spectra and they were eliminated for the establishment of the equations. A total of four spectra found were eliminated from the set of samples employed in the work. The final number of samples selected was of 130, the calibration set was composed of 104 samples and was used for the development of the different calibration equations; the external validation set was formed by 20% of the total samples (*n* = 26) and was used to evaluate the prediction capacity of each of the equations developed. The external validation set samples were selected by taking the list of samples ordered on the basis of their H values, choosing 1 of each of the five samples on the list [33]. In this way, the samples selected represented all the variability in the whole of the population [36]. To develop the calibration equations, the method of regression by modified minimum partial least squares (MPLS) was applied. The usefulness of this method has been demonstrated for the evaluation of fibre content, using the whole spectrum range (400–2500 nm) [17,27].

The spectrum correction procedure SNV-DT was applied. The latter provides the WinISI software for the elimination of dispersion due to the effects caused by the differences in particle size or the variation in length, halfway between the dispersion of the samples and fitting the baseline [37]. The treatment selected for one parameter in a dataset is not always the best option for the same parameter in any other set of samples [24]; this confirms the importance of optimizing the treatment for each parameter and dataset. In this sense, the mathematical treatments selected and applied to the spectra in our work were (0, 0, 1, 1), (1, 4, 4, 1) and (2, 5, 5, 2), in which: the first number indicates the order of the derivative (first or second derivative of the logarithm of 1/R); the second number is the amplitude or distance between the segments to be subtracted; the third number is the length of the segment to be smoothed; and the fourth number indicates a second smoothing [38]. The statistics defining the calibration equations obtained are the coefficient of determination (*R*^2^) which shows the percentage of the variability in the ADF concentrations explained by the regression equation, and the standard error of calibration (SEC), which is the standard error in the residuals for the calibration set. It should be noted that the standard error in the calibration only advises one of the fitting of the reference values to the regression line, so that it cannot be considered as being an adequate statistic for assessing the validity of the calibration equation obtained [34].

### 2.4. Equation Validation

To evaluate the prediction capacity of the calibration equations, two validation models were used, permitting the establishment of a comparison (through different statistical criteria) between the true value (obtained by the reference method) and the estimated one (obtained by NIRS).

#### 2.4.1. Cross Validation 

A cross validation was made based solely on the data employed at the calibration stage, in order to calculate the optimal number of terms in the regression. The algorithm selects different calibration and validation sets within the whole population considered, making with each selection a simulation of the regression algorithm [33,35]. Finally, the calculation software chose the equation which made the minimum standard error of cross validation (SECV). The statistics resulting from the cross validation were: the coefficient of determination of cross validation (r^2^_cv_), the standard error of cross validation (SECV), which represents the standard error of the residuals for the cross validation set; and the statistic (RPD) [2] which is the ratio between the standard deviation and the standard error of cross validation (SD/SECV). The RPD_cv_ is a statistic which permits the evaluation of the SECV in terms of the standard deviation of the reference data for the population being studied [39]:

(2) $$\mathrm{RPD}=\mathrm{SD}{\left\{{\left[\left(\sum_{i=1}^{n}{\left(y_{i}-\hat{y_{i}}\right)}^{2}\right){\left(n-k-1\right)}^{-1}\right]}^{0.5}\right\}}^{-1},$$

where y_i_ = laboratory reference value for the sample; $\hat{y_{i}}$ = NIR mean value; *n* = number of samples, *k* = number of wavelengths used in an equation; SD = standard deviation of the chemical data.

#### 2.4.2. External Validation 

The calibration equations selected with samples which did not intervene in the calibration (validation set, *n* = 26 in our work) were evaluated. The external validation statistics include: the coefficient of determination of validation (*r*^2^_ev_), the standard error of prediction (SEP), the RPD_ev_ (which is the ratio SD/SEP), and the RER [3], which is the ratio between the range of ADF of the validation as a whole and the standard error of prediction:

(3) $$\mathrm{RER}=\mathrm{range}{\left\{{\left[\left(\sum_{i=1}^{n}{\left(y_{i}-\hat{y_{i}}\right)}^{2}\right){\left(n-k-1\right)}^{-1}\right]}^{0.5}\right\}}^{-1},$$

where y_i_ = laboratory reference value for the sample; $\hat{y_{i}}$ = NIR mean value; *n* = number of samples, *k* = number of wavelengths used in an equation; SD = standard deviation of the chemical data.

The RPD and RER statistics permitted a comparison of the performance of the model through populations with different standard deviations [18]. The best calibration equations for the ADF analysis were selected by considering the optimal combination of the following external validation statistics: high values of coefficients of determination (*r*^2^_ev_) and high RPD_ev_ and RER values. Those equations in which RPD is higher than 3 were considered to have an excellent prediction ability, those with RPDs of between 2 and 3 allowed approximate predictions to be made, and those whose RPD was between 1.5 and 2 could only be used for classification purposes in groups with a high-medium-low content. Similarly, the RER values obtained with the different calibration equations with a good prediction capacity should be over 10 [39,40].

The standard error of laboratory (SEL) for the ADF analysis was determined and compared with the SEP for all the equations. To obtain the total error of the reference method (SEL), 10 samples of turnip tops and turnip greens were selected and analysed in duplicate at different times and by different analysts. The statistical ratio SEP/SEL permitted the NIRS error to be related to the error in the reference method.

## 3. Results and Discussion

### 3.1. ADF Reference Analysis in Samples of Turnip Tops and Turnip Greens 

A collection of 134 samples of *Brassica rapa* were analysed (63 turnip greens and 71 turnip tops) by the Goering and Van Soest method [7]. The mean ADF content in turnip greens and turnip tops was 11.53% and 15.98%, respectively (Table 1). A *t*-test, showed significant differences between the means. (*p* < 0.001). 

**Figure 2:**
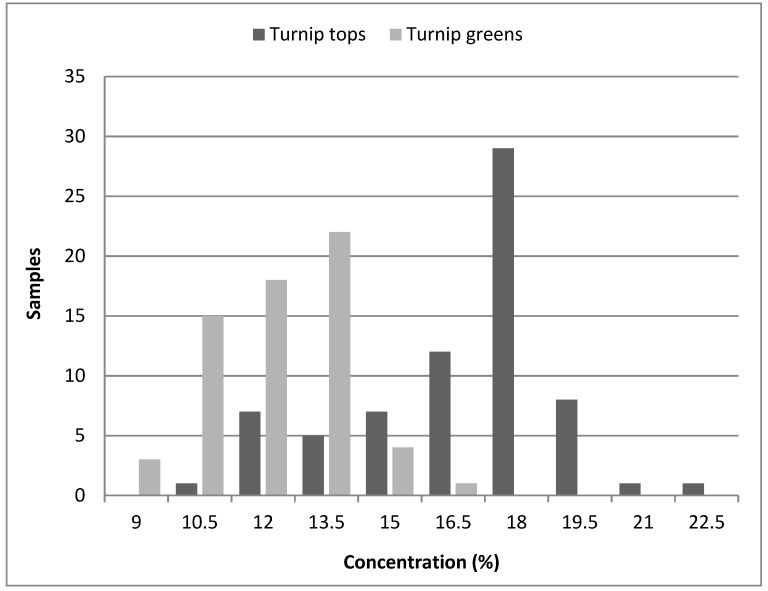
Distribution plot for ADF content in turnip greens and turnip tops.

The differences in the ADF content between turnip greens and turnip tops samples can be explained by the fact the turnip greens are formed by young leaves and the turnip tops by flower stems with a higher content of fibre. Therefore, we can conclude that the maturity of plants and the increase in structural carbohydrates lead to higher accumulation of fibre amounts in turnip tops when compared to turnip greens. These results highlight that *Brassica rapa* was a good source of fibre with high concentrations in some samples (21.91%) and lower concentrations in others (8.55%). Figure 2 shows the distribution of the frequency of fibre content in turnip greens and in turnip tops from the samples studied.

The variability in the ADF content in the samples analysed in this work was similar to that published in others studies, in which ADF values present in leaves of *Brassica rapa* were 23.50% [41]; in crude fibre 12.9% and in ADF 23.5% [42]. Previous works on the fibre content in turnip (the thickened hypocotyls of *B. rapa* widely used in human nutrition) have found values of 11.20% [41] and 14.68% [43]. In rapeseed flour the ADF content values were comprised between 9.5% and 15.2% [44]; and in seeds of other *Brassica* their values ranged from 5.33% (*B. carinata*) to 16.31% (*B. juncea*) [27]. 

The statistical data describing the calibration and validation sets are shown in Table 2. The range of values of the set of validation samples were included within the range of the values of the calibration samples, which were required to generate a calibration model with a reliable predictive ability [45].

### 3.2. Calibration and Validation

**Figure 3:**
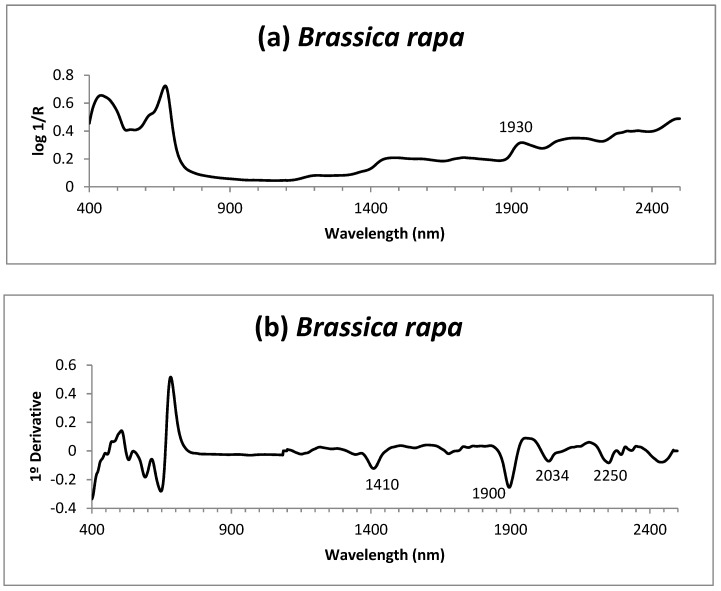

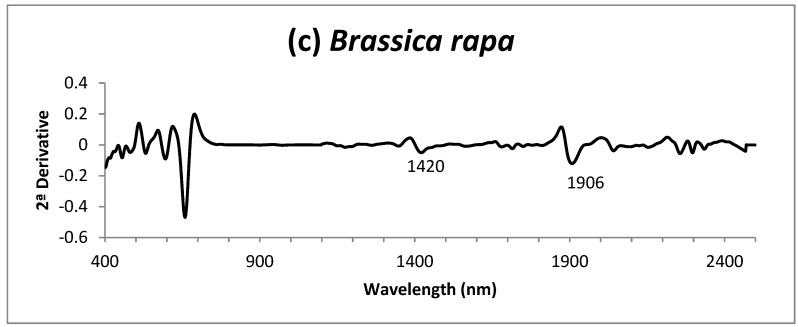
(**a**) Mean spectrum of the lyophilized green parts of *Brassica rapa*; (**b**) first derivative (SNV-DT) of the mean spectrum of the lyophilized green parts of *Brassica rapa*; (**c**) second derivative (SNV-DT) of the mean spectrum of the lyophilized green parts of *Brassica rapa*.

The principal component analysis was carried out to locate any possible spectral outliers from the calibration set [33]. Figure 3a shows the mean spectrum of the *Brassica rapa* samples in the range of 400 to 2500 nm; Figure 3b depicts the first derivative spectrum (1, 4, 4, 1; SNV-DT) and Figure 3c the second derivative spectrum (2, 5, 5, 2; SNV-DT), both derived with the application of a spectra correction treatment.

**Figure 4:**
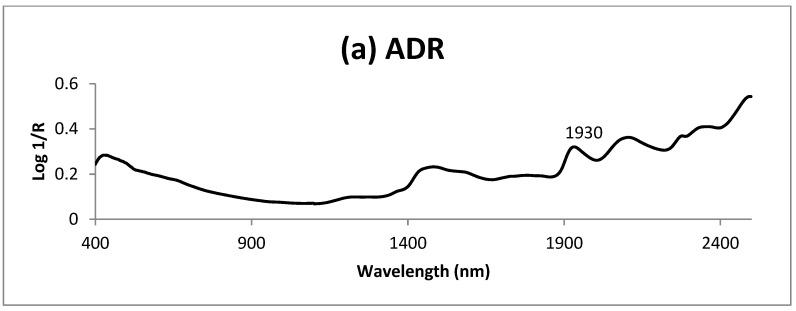

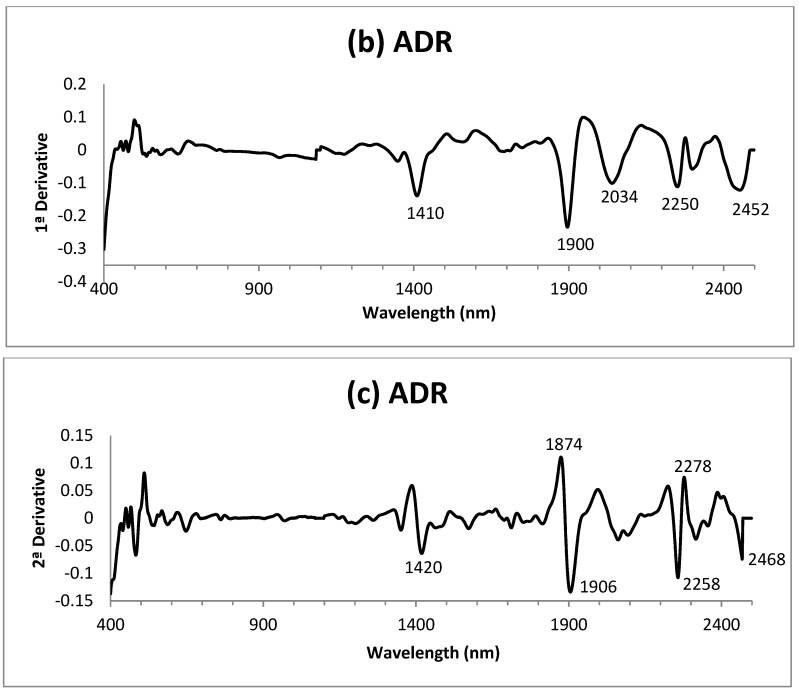
(**a**) Mean spectrum of the acid detergent residue of ADF; (**b**) first derivative (SNV-DT) of the mean spectrum of ADR; (**c**) second derivative (SNV-DT) of the mean spectrum of ADR.

With the aim of identifying wavelengths and associate spectrum bands with the ADF contained in the sample, the acid detergent residue spectrum (ADR), Figure 4, was compared with the spectrum of the green parts of *Brassica rapa*, Figure 3, in order to identify the NIRS spectrum regions which might be more related to the ADF content in the sample. In the spectra of *B. rapa* and ADR, Figure 3 and Figure 4, absorption similarities were found in certain wavelengths. It is worth noting that the wavelengths of 1420 nm related to aromatic groups, 1906 nm related to groups OH, C = O and CO_2_H and 2278 nm related to groups CH and CH_2_ associated with the structural polysaccharides of the plants, 2468 nm related to groups CH, CH_2_ and C-N-C associated with proteins [46] (WinISI II v1,50 software). Those wavelengths would participate more highly in the development of robust calibrations for the ADF content.

The results of the calibration equations obtained by MPLS regression with the three mathematical treatments is shown in Table 3. In the evaluation of the treatments applied in the development of those equations, a clear difference was found between the statistics values obtained in the equations without treatment (0, 0, 1, 1) (*R*^2^ = 0.91) and the equations with treatments with derivative the value of R^2^ = 0.95 in the first derivative (1, 4, 4, 1; SNV + DT) and a value of *R*^2^ = 0.96 in the second one (2, 5, 5, 2; SNV + DT), with both values being very similar to each other (Table 3).

#### 3.1.1. Cross Validation

On the basis of the statistics obtained in the cross validation, the final calibration equations for the ADF content were selected on the premise of maximizing the *r*^2^_vc_ and minimizing the SECV. The values of RPD_cv_ of the cross validation for ADF obtained were 3.36 (for the treatment 1, 4, 4, 1) and 3.33 (for the treatment 2, 5, 5, 2). In both equations the RPD_cv_ values were higher than 3, proving the ability of the calibration equations to be used for diagnosis and investigation purposes [40].

The two derivatization treatments (1, 4, 4, 1 and 2, 5, 5, 2) successfully optimized the model getting some optimal results in the statistics values. Both two models were valid for calibration. In the study of the profile of fatty acids in milk calibration equations, the first derivative and the second derivative were developed, (1, 5, 5, 1) and (2, 5, 5, 1), and both treatments were valid to be used in the characterization of the fat content in milk [24]. Other authors have evaluated the prediction of protein and amylose in brown rice and rice bran, where five treatments were tested including first and second derivatives, and stating that two of the treatments (1, 6, 6, 1 and 1, 4, 4, 1) were equally valid for the development of calibration equations to predict amylose content [47].

**Figure 5:**
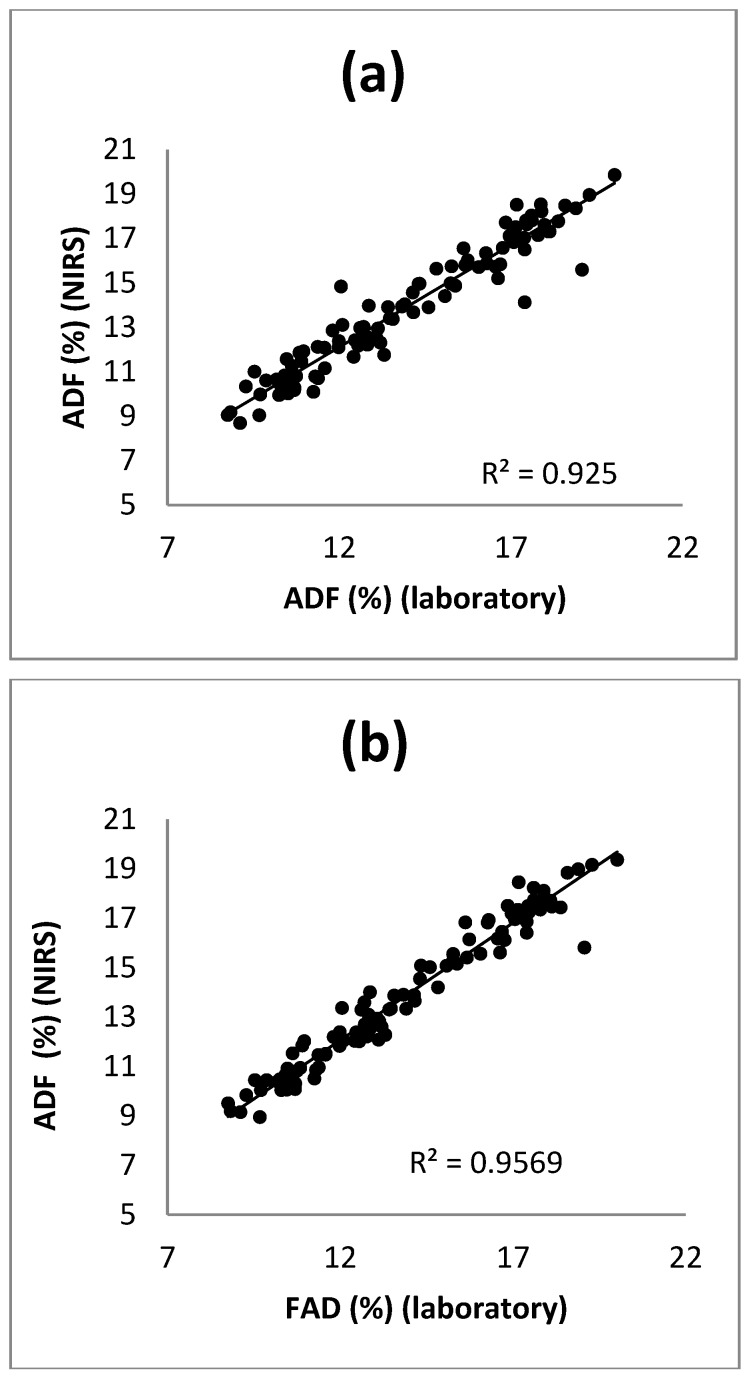
Scatter plot of the reference values against the predicted values in cross validation with respect to the ADF content applying the equations 1, 4, 4, 1 (**a**) and 2, 5, 5, 2 (**b**).

Figure 5 depicts the laboratory values compared to the NIRS prediction ones of the cross validation as a whole for ADF content.

#### 3.1.2. External Validation

Once the equations were obtained and the cross validation was performed, a second evaluation of the equations was made by using the samples not included in the calibration (external validation set) for the prediction of the ADF content. Table 4 presents the statistics of the external validation obtained for the ADF equations developed with the three mathematical treatments. In treatment 0, 0, 1, 1, a value of *r*^2^_ev_ = 0.87 was obtained, which led to lower RPD_ev_ and RER values than those of the treatments with derivatives. In treatments (1, 4, 4, 1) and (2, 5, 5, 2) the same high value in the coefficients of determination of the prediction, (*r*^2^_ev_ = 0.91) was obtained. The RPD_ev_ values were very similar to each other, 3.41 in the first derivative and 3.10 in the second one. Those results were very similar to those of RPD_cv_ obtained in the cross validation (values of over 3) and they confirmed the excellent ability to predict ADF content by using both the equations developed with treatments (1, 4, 4, 1) and (2, 5, 5, 1) [39,40]. Finally, the RER statistic was calculated and values greater than 10 were obtained in both cases: 11.79 (1, 4, 4, 1) and 11.03 (2, 5, 5, 2). This was an additional proof of the high predictive ability of the calibration models developed for ADF [39,40].

No calibration equations of the ADF content in green parts of *Brassica rapa* have been described in the bibliography up to now. However, some equations developed for the ADF content in leaves of woody species have been reported with higher values than those described (RPD_ev_ = 5.3), possibly due to the heterogeneity in the samples selected for the development of the calibration, in which different woody species collected on different dates were included [48]. ADF calibration in corn plants gave RPD_ve_ values of 2.9 [49], and other works investigating grasses leaves and red clover presented RPD = 3.4 values in NDF, which were similar to those found in this work with ADF [50].

Regarding calibrations within the genus *Brassica*, the values obtained in the calibration equations in our work are the highest described to the moment for ADF content, compared to the equations developed for ADF content in seeds found in the literature. To summarized, calibrations in intact *Brassica napus* seeds were described, with RPD_cv_ values of: 2.13 and 2.20 (in a volume of 10 mL of seed) and values of 1.91 and 2.34 (in a volume of 1 mL of seed) [16,29]; these results coincide with those of other authors who also obtained values of 1.92 in seeds of the same species [28]. As for the external validation results, those obtained in our work were also higher than those found in the literature in *B. napus* seeds, with RPD_ev_ of 2.2 and RER of 10.03 [28].

To evaluate the precision of the equations the reference method error (SEL) was calculated and was related to the SEP. The SEL value obtained was of 0.25. The SEP/SEL ratio shown in the ADF was of 4.56 in the treatment 0, 0, 1, 1, which indicates a poor precision, and values of 3.48 and 3.72 were obtained for the treatments of 1, 4, 4, 1 and 2, 5, 5, 2, respectively, which reveal a good precision; those values are similar to the ones obtained by other authors in other *Brassica* species [27].

### 3.3. Modified Partial Least Squares Loadings of the Lyophilized Green Parts Model

**Figure 6:**
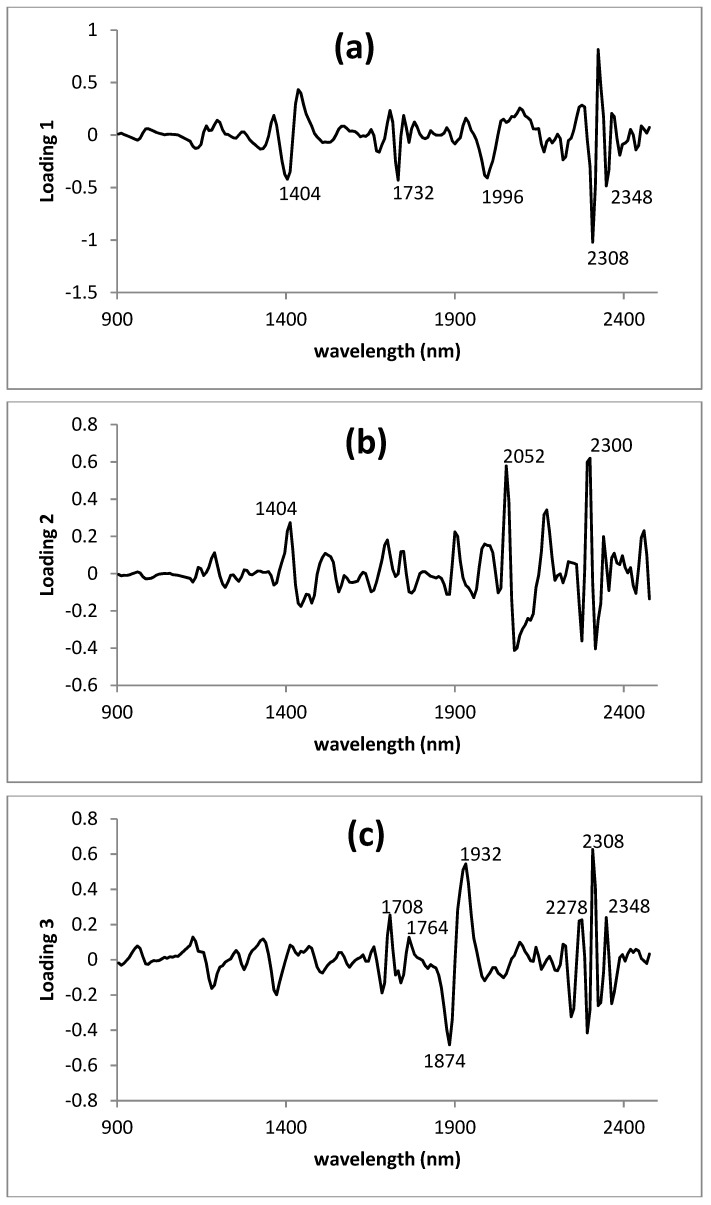
MPLS loading spectra for ADF in *Brassica rapa* in the second derivative (2, 5, 5, 2) transformations. Panels (**a**), (**b**) and (**c**) represent loadings for factors 1, 2 and 3, respectively.

Panels a, b, and c of Figure 6 represent MPLS loading spectra for factors 1, 2, and 3, respectively. These plots show the regression coefficients of each wavelength to ADF for each factor. Wavelengths represented here as participating more highly in the development of each factor are those of a greater variation and with a higher correlation with the ADF in the calibration set. In the second derivative, peaks pointing downwards indicate the positive influence of absorbers on the development of the equations, while peaks pointing upwards evidence negative correlations. Factors 1 and 3 of the lyophilized green parts model showed those most highly correlated with ADF, presenting a loading with major positive correlations at 1404, 2308 and 2348 nm, associated with the absorbance of C–H and C–O groups of lipids (Figure 6a) [27,29,46]. Factor 1 was also influenced by groups N–H at 1996 nm. Factor 2 was the one most highly correlated with amide groups in the protein region at 2052 and 2300 nm. Factor 3 was also influenced by water, as indicated by the band at 1932 nm. Wavelengths for specific absorbance of oil functional groups are known as being major contributors to NIRS calibrations for ADF in *Brassica* species and for dietary fibre in high-fat cereal products [29].

On the basis of the similarities between the second-derivative transformation of the ADR spectrum (Figure 4c) and the third MPLS loading for *Brassica rapa* (Figure 6), it seems that absorbers of the ADR participated directly in the modelling this factor, specifically, 1874 and 2278 nm related to groups CH and CH_2_ associated with the structural polysaccharides of the plants.

The study of the MPLS loadings of the ADF equation developed in this study suggests that OH groups of water, CH and CH_2_ group of structural polysaccharides, CO groups of lipids and also NH groups of amides (proteins) were the molecular associations most frequently used in modelling the equation. Shape and positioning the bands presented by the different loadings very closely resembled those reported by Font et al. [27] for oilseed *Brassicas*, in which effects due to CH groups of lipids and OH groups of water were the most important in the model. Recent NIRS calibrations for fibre fractions in intact seeds of *Brassica napus* also showed a significant contribution to the model of the CH, OH and NH groups in aromatic and protein regions [29]. 

The results obtained in the present work, both in the cross-validation and in the external validation confirm the reliability and potential of the calibration equations developed with treatments (1, 4, 4, 1) and (2, 5, 5, 2) to predict accurately and precisely the ADF content in turnip greens and turnip tops. In addition, both calibration equations (with treatments 1, 4, 4, 1 and 2, 5, 5, 2) displayed the same ability prediction of the ADF content in samples of turnip greens and turnip tops.

As a conclusion, the accurate predictions provided by the NIR equations developed in this work confirm that NIR technology could be very useful for the rapid evaluation of the ADF content in turnip greens and turnip tops. Furthermore, this technique allows us to save considerable time and money in comparison to the standard methods of analysis, making it possible to conduct large numbers of analyses for ADF content in a short time.

## Figures and Tables

**Table 1:** Fibre content in samples of turnip greens and turnip tops of *Brassica rapa*, analysed in the laboratory.

Plant Material	ADF (%)		
	Range	Mean	SD ^1^
Turnip greens (*n* = 63)	8.55–15.27	11.53	1.54
Turnip tops (*n* = 71)	10.41-21.91	15.98	2.54

^1^ SD = Standard deviation; *n* = number of samples.

**Table 2:** ADF content in the turnip greens and turnip tops samples from the calibration and validation set of *Brassica rapa* analysed following the reference method.

Sample Groups	ADF (%)		
	Range	Mean	SD ^1^
Calibration set (*n* = 104)	8.75–20.02	13.87	2.98
Validation set (*n* = 26)	8.55–18.81	13.67	3.01

^1^ SD = Standard deviation; *n* = number of samples.

**Table 3:** Calibration and cross validation statistics for ADF content in *Brassica rapa*.

		Calibration					Cross Validation		
TM ^1^	Range	Samples	Mean	SD ^2^	SEC ^3^	*R* ^2^ ^4^	SECV ^5^	RPD_cv_^6^	*R* ^2^_cv_^7^
0, 0, 1, 1	8.75–20.02	101	13.81	2.95	0.86	0.91	1.07	2.77	0.87
1, 4, 4, 1	8.75–20.02	104	13.80	2.96	0.65	0.95	0.88	3.36	0.91
2, 5, 5, 2	8.75–20.02	103	13.82	2.95	0.56	0.96	0.89	3.33	0.91

^1^ Mathematical treatment of the spectra. ^2^ Standard deviation. ^3^ Standard error of the calibration. ^4^ Coefficient of determination of the calibration. ^5^ Standard error of the cross validation. ^6^ Relation between the standard deviation and the standard error of the cross validation. ^7^ Coefficient of determination of the cross validation.

**Table 4:** Statistics of the external validation (*n* = 26) applied to the calibration equations of the fibre content in *Brassica rapa.*

TM ^1^	Range	Samples	Mean	SD ^2^	SEP ^3^	*r* ^2^_ev_^4^	RPD_ev_^5^	RER ^6^
0, 0, 1, 1	8.55–18.81	26	13.67	3.13	1.14	0.87	2.75	9.00
1, 4, 4, 1	8.55–18.81	25	13.55	2.96	0.87	0.91	3.41	11.79
2, 5, 5, 2	8.55–18.81	25	13.55	2.89	0.93	0.91	3.10	11.03

^1^ Mathematical treatment. ^2^ Standard deviation of the reference data of the external validation set. ^3^ Standard error in the prediction ^4^ Coefficient of determination of the external validation. ^5^ Relation between the standard deviation and the standard error in the prediction. ^6^ Relation between the data range and the standard error in the prediction.
